# Effects of Hyperoxia on Oxygen-Related Inflammation with a Focus on Obesity

**DOI:** 10.1155/2016/8957827

**Published:** 2015-12-01

**Authors:** Pedro González-Muniesa, Laura Garcia-Gerique, Pablo Quintero, Suyen Arriaza, Amaya Lopez-Pascual, J. Alfredo Martinez

**Affiliations:** ^1^Centre for Nutrition Research, Department of Nutrition, Food Sciences and Physiology, University of Navarra, Irunlarrea 1, 31008 Pamplona, Spain; ^2^Centro de Investigación Biomédica en Red Fisiopatología de la Obesidad y Nutrición (CIBERobn), Instituto de Salud Carlos III, Madrid, Spain; ^3^Navarra's Health Research Institute (IDISNA), 31008 Pamplona, Spain; ^4^Departamento de Gastroenterología, Facultad de Medicina, Pontificia Universidad Católica de Chile, Libertador Bernardo O'Higgins 340, Santiago, Chile

## Abstract

Several studies have shown a pathological oxygenation (hypoxia/hyperoxia) on the adipose tissue in obese subjects. Additionally, the excess of body weight is often accompanied by a state of chronic low-degree inflammation. The inflammation phenomenon is a complex biological response mounted by tissues to combat injurious stimuli in order to maintain cell homeostasis. Furthermore, it is believed that the abnormal oxygen partial pressure occurring in adipose tissue is involved in triggering inflammatory processes. In this context, oxygen is used in modern medicine as a treatment for several diseases with inflammatory components. Thus, hyperbaric oxygenation has demonstrated beneficial effects, apart from improving local tissue oxygenation, on promoting angiogenesis, wound healing, providing neuroprotection, facilitating glucose uptake, appetite, and others. Nevertheless, an excessive hyperoxia exposure can lead to deleterious effects such as oxidative stress, pulmonary edema, and maybe inflammation. Interestingly, some of these favorable outcomes occur under high and low oxygen concentrations. Hereby, we review a potential therapeutic approach to the management of obesity as well as the oxygen-related inflammation accompanying expanded adipose tissue, based on elevated oxygen concentrations. To conclude, we highlight at the end of this review some areas that need further clarification.

## 1. Introduction

Obesity is caused by an imbalance between energy intake and energy expenditure that results in an enlarged growth in adipose tissue that is generally harmful to health [1]. This burden of obesity on health extends across multiple organ systems and diseases [2] since excessive fat deposition is related to a higher prevalence of cardiovascular disease, metabolic syndrome features, different type of cancers, and other adverse clinical conditions [3]. In addition, obesity has been associated with higher mortality rates [4].

In the last half century, the prevalence of human obesity has risen dramatically all over the world [5]. High-income countries are not the only ones affected by the epidemic, as the condition is achieving alarming rates in the transition world as well [6]. Thus, it has been reported that prevalence of obesity has almost doubled from 6.4% in 1980 to 12.0% in 2008 in the entire world. Half of this rise occurred from 2000 to 2008 [7]. Furthermore, during 2013 worldwide obesity prevalence was estimated at 36.9% in men and 38% in women, while obesity-associated mortality and treatment expenses make this disease the major global health challenge [8]. In addition to unhealthy habits (consumption of high-energy yielding foods and low physical activity), the interaction with genetic factors could be involved in this increased prevalence [9]. Although human genome cannot change in such short time, mechanisms involving epigenetics have been proposed as a possible origin and/or development of this increase [10]. Other factors have been suggested, such as microbiota, increasing maternal age, greater fecundity among obese people, assortative mating, sleep deprivation, endocrine disruptors, pharmaceutical iatrogenesis, reduction in variability of ambient temperatures, and intrauterine and intergenerational effects [11].

At the tissue level, obesity is known to provoke a mild but chronic inflammation state within the adipose tissue, leading to multiple metabolic disorders if the condition persists [2]. Among the features that may lead to this inflammatory response in obesity, it has been hypothesized that poorly oxygenated adipose tissue may underlie the initiation and development of this process [12, 13], although the relationship between tissue oxygen partial pressure and adipose tissue inflammatory process is still under debate [14–16]. Regarding hypoxic conditions, several human studies have related environmental hypoxia during expeditions at moderate- and high-altitude conditions to a reduction of food intake [17, 18], subsequent metabolic alterations, and weight loss [19, 20]. Similarly, our group found that rats exposed to normobaric hypoxia reduced their food intake and, consequently, their weight [21]. It is important to highlight that this hypobaric hypoxia differs from the hypoxia described in obese adipose tissue [22].

## 2. Inflammation and Obesity

Inflammatory processes are complex biological responses orchestrated by tissues to combat injurious stimuli, as host defense, tissue remodeling, and metabolic changes, in order to maintain cell homeostasis [23]. More precisely, the inflammatory phenomena involve multiple specific cell processes such as recruitment and activation of immune cells (leukocytes, granulocytes, monocytes, lymphocytes, and dendritic cells), stimulation of the production of different chemical bioactive mediators (such as cytokines, chemokines, or prostanoids), regulation of signaling pathways involving insulin, leptin, glucose, or lipids, and eventually epigenetic regulation of the expression of important related genes as nuclear factor kappa-light-chain-enhancer of activated B cells (*NF-κB/AP1*) activity or* IL-6* expression [24–26]. This adaptive response can be instantaneous and short, which is known as acute inflammation, or long and gradual as in a chronic, mild inflammatory process. The latter has been established as a main cause and/or a consequence of diverse diseases that may need pharmacological interventions to attenuate the cellular inflammatory routes such as diabetes, cardiovascular diseases, and obesity [27].

Obesity itself is characterized by a state of mild chronic inflammation in humans [28]. In fact, obesity-induced inflammation has been described as metaflammation, that is, a chronic and low-grade inflammatory response initiated by excess nutrients in metabolic cells, where circulating levels of an important number of inflammatory markers, such as C-reactive protein (CRP), haptoglobin, Interleukin-1 (IL-1), Interleukin-6 (IL-6), monocyte chemoattractant protein-1 (MCP-1), plasminogen activator inhibitor-1 (PAI-1), or tumor necrosis factor-alpha (TNF-*α*), are increased [1, 29, 30]. Additional studies suggest that adipose tissue inflammatory state is orchestrated by monocyte/macrophage infiltration and overproduction of proatherogenic cytokines, two situations that are related to the initiation and development of several obesity-associated diseases such as atherosclerosis and cardiovascular complications [31–33]. Indeed, about half of the cells that make up adipose tissue of obese mice were identified as macrophages, a feature that was associated with low-grade systemic inflammation [33]. At this point, it is important to note that there is a very specific phenotype called “Metabolically Healthy Obese (MHO),” which presents the following features: less visceral fat, less infiltration of macrophages into adipose tissue, and smaller adipocyte cell size, leading to a more favorable inflammatory profile, although a recent meta-analysis has reported that these subjects are at an increased risk of developing type 2 diabetes compared with their age-matched lean controls [34]. In this sense, diet has a main role in the inflammation [35, 36] and oxidative stress-related outcomes [37, 38].

**Figure 1:**
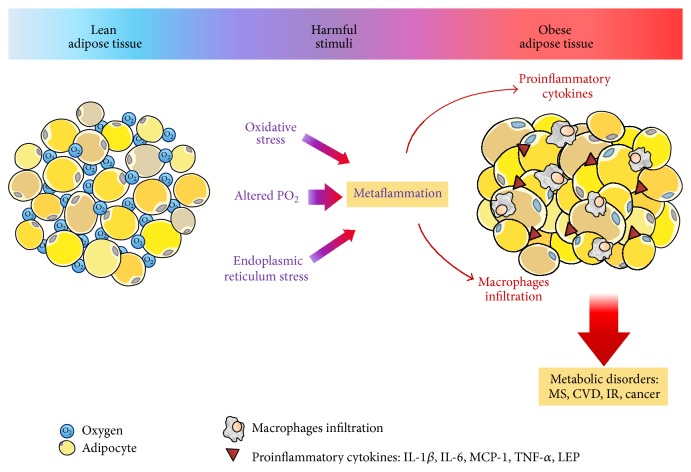
Effects of obesity on adipose tissue. Oxidative stress, altered PO_2_, and endoplasmic reticulum stress are potential triggering factors for metaflammation development. This chronic low-grade inflammation is associated with proinflammatory adipokines release and the concomitant macrophages migration to metaflammation zone. And, then, positive feedback for proinflammatory signals is perpetuated in the tissue which could be related to posterior metabolic disorders. IL-1*β*: Interleukin-1*β*; IL-6: Interleukin-6; TNF-*α*: tumor necrosis factor-*α*; LEP: leptin; MS: metabolic syndrome; CVD: cardiovascular disease; IR: insulin resistance.

Certainly, several signaling pathways have been proposed to explain the cause for the initiation of inflammatory processes during obesity (Figure 1), including oxidative stress [39], endoplasmic reticulum stress [40], and adipose tissue hypoxia [13]. These features may provoke the release of reactive molecules that can interact with proteins, lipids, or DNA, causing cell damage/death and leading to nonspecific proinflammatory effects [41]. These theories can explain some aspects of inflammation and metabolic disorders in obesity, but the link between obesity and these factors still remains elusive [13, 42].

## 3. Hypoxia and Inflammation in Adipose Tissue

Adipose tissue is constituted not only by adipocytes, but also by a stromavascular cell fraction, which involves leukocytes (including macrophages), T cells, and natural killer cells [43]. Some of these cells are a dominant source of inflammatory cytokines and are, therefore, appropriate targets for the study of mechanisms underlying hypoxia-induced inflammation within this tissue [1, 44]. Inflamed tissues are often characterized by decreased oxygen availability and cells must be able to maintain viability and proper function in strongly variable microenvironments [45].

**Figure 2:**
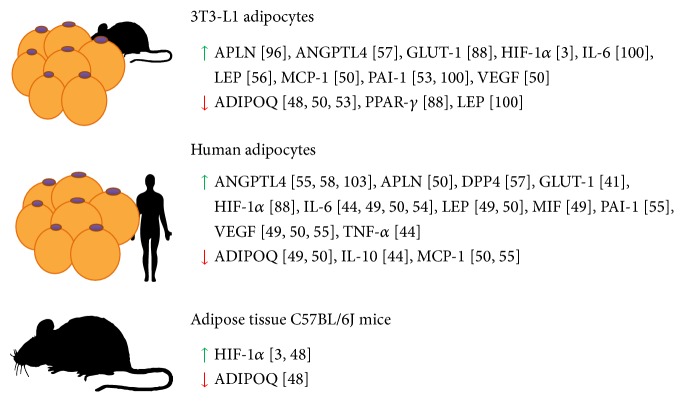
Effects of hypoxia on the secretion of key proteins in mice and human adipocytes and in adipose tissue of C57BL/6J mice (green up arrow) increased and (red down arrow) decreased protein in response to hypoxia). ADIPOQ: adiponectin; ANGPTL4: Angiopoietin-like protein 4; APLN: Apelin; DPP4: dipeptidyl peptidase-4; GLUT-1: Glucose Transporter 1; HIF-1*α*: hypoxia-inducible factor-1*α*; IL-6: Interleukin-6; IL-10: Interleukin-10; LEP: leptin; MIF: macrophage migration inhibitory factor; MCP-1: monocyte chemoattractant protein-1; PAI-1: plasminogen activator inhibitor-1; VEGF: vascular endothelial growth factor; TNF-*α*: tumor necrosis factor-*α*.

Indeed, hypoxia has been proposed as a key initiator of adipokine dysregulation in obesity [22] by inducing the expression of certain genes in adipocytes and macrophages like* TNF-α, IL-1, IL-6, MCP-1*, and* PAI-1*, macrophage migration-inhibition factor (*MIF*), inducible-nitric oxide synthase (*iNOS*), and others [41, 46–48]. Similarly, the production of vascular endothelial growth factor (VEGF) inducing a proangiogenic response is stimulated by hypoxia in human and murine adipocytes [49, 50]. Furthermore, the role of hypoxia and angiogenesis in tumor progression has been described in several studies [51, 52]. In addition, it has been reported that low O_2_ availability may change the expression of diverse proinflammatory molecules [44, 50, 53–56] and, also, inhibits enzymes related to lipid metabolism such as Lipoprotein lipase by upregulating angipoietin-like protein 4 (Angptl4) [57]. Nonetheless, different behavior patterns are found depending on cellular origin, for example, human or mouse (Figure 2).

Many of the aforementioned genes have been found to be dependent on the activation of hypoxia-inducible factor-1 alpha (HIF-1*α*), the master transcriptional regulator of hypoxia environment [56, 58, 59], since cellular adaptation to hypoxia appears as a defense mechanism adopted by cells to conserve the optimum oxygen concentration required for vital metabolic functions [41], where activation of specific and important transcription factors, such as HIF-1 or NF-*κ*B, occurs [60]. The transcription factor HIF-1 is a heterodimer composed of an oxygen sensitive subunit HIF-1*α* and a constitutively expressed subunit HIF-1*β* [41, 61, 62]. This protein is responsible for the induction of genes that facilitate accommodation and survival from hypoxic stress [63]. When cellular oxygen levels are normal, this protein is immediately targeted for proteasome degradation [3]. However, under lower oxygen concentrations [64] and mild oxidative stress (induced with H_2_O_2_) [65], as in inflamed tissues, hydroxylation is inactivated, enabling the binding of CREB-Binding Protein (CBP/p300) coactivator, and therefore HIF-1*α* becomes stable, leading to the expression of HIF-1 target genes. Several studies have described an increase in* HIF-1α* expression and protein levels in the adipose tissue of dietary obese mice and ob/ob obese mice, which are thought to suffer a low oxygen supply [3, 48]. Moreover, human primary preadipocyte cultures have shown higher levels of* HIF-1α* mRNA and protein than mature adipocytes [49]. These results suggest a posttranslational regulation, where HIF-1*α* protein increases independently of mRNA level in response to hypoxia in adipose tissue. Interestingly, in human primary adipocytes, an elevation of* HIF-1α* mRNA and protein levels has been described after 8 and 24 h of hypoxic exposure [50]. Other studies observed an enhancement of* Hif-1α* mRNA and protein levels in 3T3-L1 cells during adipogenesis [3, 66], whereas mRNA levels of* Hif-1α* did not change in an assay performed in 3T3-L1 cells under these conditions [46].

Although these changes are consistent with hypoxia, they are not necessarily a direct response to low oxygen concentrations, since other factors such as reactive oxygen species (ROS) production and/or endoplasmic reticulum stress are likely involved [1]. Furthermore, another scientific group has suggested that, despite lower adipose tissue blood flow, human adipose tissue may suffer hyperoxia in obese subjects, explained apparently by lower oxygen consumption within this organ [15], although this needs to be further elucidated.

## 4. Treatments with Hyperoxia

Hyperoxia is referred to as a potentially harmful major lifesaver [67]. Thus, oxygen is used in current medicine as a treatment for several diseases such as chronic obstructive pulmonary diseases, management of ulcers in diabetic patients, and cerebral ischemia wounds and hypothesized and studied as a tool for weight management [46, 68, 69]. Nevertheless, an excess of the exposure to oxygen in time and/or concentration may lead to important deleterious effects [70, 71].

Hyperoxia increases the amount of dissolved oxygen in blood with the subsequent action on tissue oxygenation and mitochondrial metabolism [72]. These mechanisms explain why oxygen is used as a therapy to optimize oxygen transport capacity [73]. There are two clearly defined therapies commonly used at the clinical level: hyperbaric oxygen therapy (HBOT) and normobaric oxygen therapy (NBOT). The former involves the inhalation of 100% O_2_ in a chamber at pressure greater than at sea level [74], while the latter involves administering more than 21% O_2_ at normal atmospheric pressure.

Over the past years, multiple studies have documented NBOT and HBOT to have important clinical applications (see the following).

Experimental and nonapproved claimed beneficial effects of hyperoxia treatment are as follows:

 Anti-inflammatory effect [75].
 Better control of blood glucose levels [76].
 Cytoprotection in chemotherapy side effects [77].
 Decreased inflammatory pain [78].
 Decreased ischemic stroke mortality and comorbidities [79–81].
 Enhanced survival and regeneration of fat grafting [82].
 Improved glucose utilization by the brain [83].
 Increased angiogenesis [84].
 Increased insulin sensitivity [85].
 Neuroprotection in hypoxic-ischemic injury [86, 87].
 Neuroprotection in traumatic brain injury [88].
 Terminated migraine headache pain [89].
 Tumour control and decreased mortality and recurrence [90].
 Wound healing [91–94].

For example, oxygen therapy has demonstrated effectiveness in the treatment of acutely ischemic brain tissue, in acute ischemic stroke, in the amelioration of pathological brain infarct volumes, and in the enhanced survival and regeneration of fat grafting, among others [71, 79–82, 95, 96]. The acting mechanisms in this scenario have been attributed, in part, to an increase in the aerobic metabolism and angiogenesis processes, leading to an improvement of the natural vascularization phenomena [84]. Moreover, several investigations have shown the beneficial effects of HBOT on wound healing [91–93], relieving migraine headaches [90], and local tumour control, mortality, and recurrence for cancers of the head, neck, and uterine cervix [89, 90].

Regarding metabolic alterations, there is evidence that HBOT and NBOT are able to attenuate systemic inflammation. On a zymosan-induced generalized inflammation mouse model, 80% survival rate of mice treated with 100% O_2_ with respect to control has been reported [97]. Moreover, zymosan mice treated with 100% O_2_ showed significant reductions in the expression of inflammatory cytokines and little distortion in overall tissue architecture compared to the animals maintained in room air. Finally, O_2_ treated mice also presented significant improvements in serum alanine aminotransferase (ALT) test, aspartate aminotransferase (AST) test, and creatinine. Importantly, amelioration of inflammation and pain was also obtained in carrageenan-induced rats exposed to HBOT [78]. Taken together, these researches clearly illustrate that 100% O_2_ treatment may improve organ structure and function as well as suppress the inflammatory cascade response, suggesting the possibility of oxygen treatment to be used in chronic cases of inflammation.

Furthermore, there is evidence that HBOT reduces blood glucose levels in patients with type 2 diabetes and hypertension [76]. This beneficial outcome may be due to an increase in glucose utilization by the brain, a feature that has been documented after HBOT exposure in rats [83], while insulin has also been described to have important actions in these circumstances. Indeed, a recent study has shown that HBOT (100% O_2_; 2.0 ATA for 2 h) was able to increase insulin sensitivity in both healthy subjects and obese individuals with type 2 diabetes. Of note, this positive effect, which authors state to be equivalent to that found after moderate weight loss, was observed within 3 days of HBOT and maintained for 30 sessions [85]. However, the implicated mechanisms are unknown and require further elucidation.

Interestingly, various studies have provided evidence for an analogous beneficial effect under hypoxic conditions, on glucose homeostasis and adipose tissue inflammation, in rodents [98, 99], and on insulin sensitivity in diabetic patients [100] and obese subjects [101]. Furthermore, several studies have also reported healthier values of blood pressure for humans that have exercised under hypoxia [102–104]. Nevertheless, other researchers have found no significant differences in obese people in blood glucose levels [105] and in obese people suffering sleep apnea-hypopnea syndrome in the blood levels of glucose, insulin, and relevant inflammatory parameters [104].

In addition, Obstructive Sleep Apnea (OSA), which appears to be a direct cause and a direct consequence of weight gain [106], is benefitted by the use of hyperoxia primarily based on its ability to reduce loop gain (LG), defined as the ratio of the ventilatory overshoot to the preceding reduction in ventilation [107].

Overall, these studies evidence that oxygen exposure has also important positive effects at a systemic level, being able to ameliorate inflammation and metabolic disruptions, which are features commonly presented in obesity. Considering that oxygen treatment may also be harmful due to proinflammatory outcomes, next steps should focus on investigating the delicate balance between oxygen protection and toxicity, determining the optimal duration, partial pressure, and timing of treatment, among others. In this sense, it is interesting to note that weight reduction increases partial oxygen arterial blood pressure (PaO_2_) and, also, that morbidly obese women seem to have at rest a better gas exchange [108].

## 5. Studies Related to Hyperoxia and Its Associated Molecular Effects

Several studies have reported that high oxygen concentrations can modulate mRNA expression of several genes and related protein secretion (some genes of interest for the topic of this review are listed in Table 1). For example, clinical and experimental studies have demonstrated that increasing oxygen concentrations in hypoxic and/or ischemic wounds accelerate the healing process by increasing blood vessels growth [94]. Specifically, this study observed an increase of VEGF synthesis in wounds of Sprague-Dawley rats exposed to HBOT (100% O_2_ for 90 minutes twice daily for 7 days). Moreover, after 5 h of HBOT (90 min at 97.5% O_2_ at 2.4 ATA) nineteen genes involved in adhesion, angiogenesis, inflammation, and oxidative stress were downregulated [109]. Notably, only angiogenin gene expression (which promotes both angiogenesis and nitric oxide production) was upregulated. This situation induced a decrease in endothelial* Il-8* mRNA expression and further protein secretion, leading to an alleviation of inflammatory processes during chronic wound healing. Indeed, a study performed in mice found out that the cholinergic pathway seems to be the underlying mechanism by which the HBOT has an anti-inflammatory role [110]. On the other hand, neonatal rats exposed to NBOT had long term adverse effects related to cardiovascular and renal dysfunctions in the adulthood [111].

Regarding lung injury, a study carried out in alveolar macrophages obtained from children with interstitial lung disease observed a decrease in* TNF-α*,* IL-1,* and* IL-6* expression and an increase in Interleukin-8 (*IL-8*) expression after hyperoxia exposure [75]. These data confirmed that hyperoxia induces changes in mRNA and protein levels in macrophages. The effect of HBOT and NBOT was analysed in Sprague-Dawley rat newborns exposed to 8% O_2_ for 2 h. One hour after hypoxia exposure mice were treated with 100% O_2_ under normobaric or hyperbaric conditions. Results confirmed that a single administration of HBOT or NBOT dose dependently reduced the hypoxic-ischemic-induced elevation of HIF-1*α* [86]. Indeed, oxygen regulates the degradation of HIF-1*α*, and the HIF-1*α*-depending gene regulation is responsible for several different genetic expressions such as erythropoietin (*EPO*) and* VEGF*. These genes are frequently expressed in parallel, leading to the possibility that HIF induction could stimulate immune response by inflammatory cells [122]. For example, after hyperoxia treatment, VEGF downregulation could decrease tumour angiogenesis, and the induction of* EPO*-expression could provide cytoprotection, processes that could be deleterious for cancer cells while helping nonmalignant cells (at least neural and cardiac) to be protected from the side effects of chemotherapy [77]. Nonetheless the influence of HBOT on* HIF* isoforms expression in other cell types or tissues is variable [123]. In this regard, no changes were observed in* Hif-1α* gene expression modifications in 3T3-L1 adipocytes exposed to 95% O_2_ [46]. Thereby further experiments need to be performed, always bearing in mind that HIF-1*α* mRNA expression is particularly unstable. Therefore, data related to HIF-1*α* mRNA expression should be interpreted with prudence.

Concerning O_2_ treatment and adipose tissue, our group was the first to evaluate the effect of hyperoxia (95% O_2_; 24 or 48 h) on 3T3-L1 adipocytes. In these experiments, a strong proinflammatory response was observed, as demonstrated by the release of intra- and extracellular ROS and the upregulation of proinflammatory adipokines such as* Il-6* and* Mcp-1* [46, 69]. A strong correlation between* Mcp-1* mRNA expression and ROS release was also found [46], a result that is in accordance with other studies showing that ROS production could increase* Mcp-1* expression [124]. However, other interesting outcomes were observed, such as upregulation of peroxisome proliferator-activated receptor gamma (*Ppar-γ*) signalling [46]. This finding is in agreement with the above-mentioned studies linking O_2_ therapy and amelioration of insulin sensitivity and suggests that adipose tissue may also contribute to this feature. Finally, hyperoxia caused a decrease in the expression of Angiopoietin-like 4 (*Angptl4*) [46, 69], which is a protein that regulates plasma triacylglycerides metabolism by inhibiting lipoprotein lipase [125]. In this context, some authors have proposed that elevated* ANGPTL4* expression might be involved in hypertriglyceridemia in patients with insulin resistance [126] and other hypoxic conditions [103]. Thus, a downregulation in* Angptl4* expression, as it occurs with hyperoxia in 3T3-L1 adipocytes, might contribute to ameliorating these metabolic disorders. Furthermore, glycerol and lactate release were increased and decreased, respectively, under an elevated oxygen exposure in 3T3-L1 adipocytes [46, 69], and in male Wistar rats fed with control diet a similar effect on lactate was found (Pablo Quintero, Pedro González-Muniesa, and J. Alfredo Martinez, unpublished results). Lactate inhibits lipolysis in adipose tissue by mediating, through GPR81, the antilipolytic action of insulin [127], and therefore a reduction in lactate might be considered beneficial against the appearance and development of complications associated with obesity. From these experiments, it can be gathered that hyperoxia activates a pernicious proinflammatory status although it seems to have beneficial effects on glucose and lipid metabolism [46, 69]. In contrast with this, a study by Hodson et al. 2013 has reported in humans an inverse relationship between the amount of lactate released by adipose tissue and the BMI of the subject [16]. Interestingly, hyperoxia in mice leads to weight loss and an increase in leptin, an adipokine involved in the regulation of food intake, although it seems that this weight loss is not dependent on leptin [116]. Intriguingly, a similar result was found in mice after 21 days being exposed to chronic hypoxia (8% O_2_), in which these animals lose weight and adipose tissue mass and size, and leptin concentrations were decreased [101]. Nevertheless, more studies are needed to obtain a better understanding of these mechanisms and, more importantly, to determine the optimum duration and timing of treatment to avoid undesired effects. Moreover, some results are contradictory or not comparable due to the wide variety of concentrations and/or duration of the exposures and also the experimental models and analyzed tissues. For instance, the gene expression of the proinflammatory cytokine IL-8 was increased in alveolar macrophages from interstitial lung disease treated with 95% O_2_ for 48 h [109], while decreased in chronic wound of umbilical vein endothelial cells treated with 95% O_2_ for 90 minutes [75]. These data suggest that more research is needed regarding the different tissues, experimental models, and type of treatments to elucidate the benefits and disadvantages of hyperoxia therapy.

## 6. Conclusions and Future Directions

Oxygen homeostasis is of fundamental importance to the cell and to maintain equilibrium within the existing complex relationships between oxygen concentration, energy metabolism, acid-base status, redox state, and the control of cell growth and proliferation [63]. The studies herein reviewed evidenced that an abnormal level of oxygen partial pressure in expanded adipose tissue may be a triggering factor for the release of inflammatory mediators. Moreover, several studies have demonstrated that treatment with hyperoxia/hypoxia may play an important role in the regulation of inflammatory responses and metabolic disorders, such as insulin resistance. Furthermore, aerobic exercise, which increases body general oxygenation, seems to play a main role in visceral adipose tissue reduction [128].

Taking into account the information presented in this review and further preliminary experimental data, we put forward some questions in order to address future investigations about metaflammation treatment with hyperoxia exposure:

1. Which is the origin of proinflammatory signalling in obesity and its role in obesity-associated manifestations?
2. Would HIF-1*α* signalling be a possible therapeutic target in an obesity context?
3. Which are the possible effects of oxygen therapy as an obesity treatment, particularly on metaflammation?

Yet, more studies are needed to shed more light on the molecular effects of oxygen (hyperoxia or hypoxia) and the concentration available of this gas in various fat depots from humans with different phenotypes, for example, those suffering obesity with or without insulin resistance. Maybe one day this therapy could be used as an advantageous tool to improve various diseases concerning inflammatory conditions.

## Figures and Tables

**Table 1:** Results of the responses (mRNA, protein, and ROS production) to different oxygen exposures in different experimental models.

Gene	Model/tissue	Treatment	Duration	mRNA	Prot.	Authors
*Angptl4*	3T3-L1/adipocyte culture	95% O_2_	24 h	↓	ND	[46]

*Hif-1α*	3T3-L1/adipocyte culture	95% O_2_	24 h	ns	ND	[46]
	Male C56BL/6J mice/brain	50% O_2_	1 week	ns	ND	[112]
	Sprague-Dawley rat/brain with IH damage	NBOT/HBOT	2 h	ND	↑↑/↑	[86]

*Il-1*	Sprague-Dawley rat/lung	90% O_2_	10 h, on postnatal day 14	ND	↑	[113]
	Human/alveolar macrophages primary culture (ILD)	95% O_2_	48 h	↓	ND	[75]
	C57BL/6J mice/lung	>95% O_2 _	3 days	↑	ND	[114]

*Il-6*	Sprague-Dawley rat/lung	90% O_2_	10 h, on postnatal day 14	ND	↑	[113]
	3T3-L1/adipocyte culture	95% O_2_	24 h	↑	ND	[46]
	Male C57BL/6J WT mice and db/db mice/BAL	100% O_2_	84 h	ND	↑	[115]
	Human/alveolar macrophages primary culture (ILD)	95% O_2_	48 h	↓	ND	[75]
	C57BL/6J mice/lung	>95% O_2 _	3 days	ND	↓	[114]

*Il-8*	Human/UVEC culture with chronic wound	97.5% O_2_	90′	↓	↓	[109]
	Human/alveolar macrophages primary culture (ILD)	95% O_2_	48 h	↑	ND	[75]

*Leptin*	3T3-L1/adipocyte culture	95% O_2_	24 h	ns	ND	[46]
	Male C57BL/6J WT mice and db/db mice/BAL	100% O_2_	84 h	ND	↑	[115]
	Female C57BL/6 ob/ob mice/adipose tissue	100% O_2_	72 h	↑	ND	[116]

*Tnf-α*	Sprague-Dawley rat/lung	90% O_2_	10 h, on postnatal day 14	ND	↑	[113]
	Male C57BL/6J WT mice and db/db mice/BAL	100% O_2_	84 h	ND	↑	[115]
	Female 57BL/6 ob/ob mice/adipose tissue	100% O_2_	72 h	↓	ns	[116]
	Human/alveolar macrophages primary culture (ILD)	95% O_2_	48 h	↓	ND	[75]
	Sprague-Dawley rat/lung macrophages primary culture	100% O_2_	90′	ND	↑	[117]
	C57BL/6J mice/lung	>95% O_2_	3 days	ND	↑	[114]

*Vegf*	Sprague-Dawley rat/lung	90% O_2_	10 h, on postnatal day 14	ND	↓	[113]
	Male C56BL/6J mice/brain	50% O_2_	1 week	↓	ND	[112]
	Sprague-Dawley rat/wound fluid	100% O_2_	90′ twice daily for 7 days	ND	↑	[94]
	Sprague-Dawley rat/liver	95% O_2_	2 weeks, newborn	ND	↓	[118]

ROS	3T3-L1/adipocyte culture	95% O_2_	24 h	↑		[46]
	Sprague-Dawley rat/carotid body and PG/NG complex	95% O_2_	4 h	↑		[119]
	Male Sprague-Dawley rats/lung capillary endothelial cells	70% O_2_	90′	↑		[120]
	C57BL/6J mice/liver with ischemia	60% O_2_	24 h	↑		[121]

*Angptl4*: Angiopoietin-like 4; *Hif-1α*: hypoxia-inducible factor-1*α*; *Il-1*: Interleukin-1; *Il-6*: Interleukin-6; *Il-8*: Interleukin-8; *Tnf-α*: tumor necrosis factor-*α*; *Vegf*: vascular endothelial growth factor; ROS: reactive oxygen species; BAL: bronchoalveolar lavage; CIH: chronic intermittent hypoxia; ILD: interstitial lung disease; UVEC: umbilical vein endothelial cells; ND: no data; ns: no significant difference detected.
